# UV-Protective Polymers Developed by Multicomponent Reactions

**DOI:** 10.3390/polym18212719

**Published:** 2026-11-02

**Authors:** Zhuoyuan Lin, Siyu Pan, Baoqing Zhang, Lei Tao

**Affiliations:** 1 The Key Laboratory of Bioorganic Phosphorus Chemistry and Chemical Biology (Ministry of Education), Department of Chemistry, Tsinghua University, Beijing 100084, China; linzy23@mails.tsinghua.edu.cn; 2 Kingfa Science & Technology Co., Ltd., Guangzhou 510663, China; 3 Beijing Plastics Research Institute Co., Ltd., Beijing 100009, China; zhbqwork@sina.com

**Keywords:** multicomponent reactions, UV protection, polymeric UV filter, UV-protective film, UV-protective fabric

## Abstract

Excessive ultraviolet (UV) radiation does harm to human health and also degrades the mechanical properties and appearance of materials. Conventional organic UV filters suffer from poor photostability and potential biotoxicity, while inorganic nanoparticles require high loading and stable dispersing. Multicomponent reactions (MCRs) can achieve UV-resistant products that efficient absorb UV radiation through the heterocyclic structures present in the products or through the introduction of UV-absorbing groups. MCRs are efficient, atom-economical, and highly modular, providing a practical tool to develop polymers with UV-protective functionality. This mini review summarizes recent progress in UV-protective polymers prepared by MCRs, including the design concepts and synthesis of MCR-based agents that protect cells against UV-induced oxidative stress, polymeric sunscreens, multifunctional UV-protective films, and UV-protective fabrics. Additionally, we also outline the extension of this strategy to protect against other harmful radiations, such as harmful blue rays. Finally, we have a brief discussion on future perspectives of UV-protective polymers via MCRs.

## 1. Introduction

Ultraviolet (UV) rays constitute the portion of the solar spectrum with wavelengths between those of visible light and X-rays. According to wavelength range, UV radiation can be divided into three main regions, namely UVA (320–400 nm), UVB (280–320 nm), and UVC (200–280 nm), which differ in energy and penetrating capacity [1]. UV radiation exerts dual effects on living organisms. On the one hand, it promotes vitamin D synthesis in the human body [2] and inactivates pathogenic microorganisms that threaten human health [3]. On the other hand, prolonged exposure to UV radiation may induce skin photoaging [4] and even increase the risk of skin cancer [5]. For materials, UV radiation is a major cause of material fading and yellowing, as well as the deterioration of their mechanical properties [6,7,8]. Ignoring the protection against harmful ultraviolet rays will pose a high risk to human life and health as well as the performance of materials.

To mitigate or eliminate the adverse effects of UV radiation, a variety of UV-protective technologies and materials have been developed. The UV-protective performance of organic materials is typically achieved by incorporating natural or synthetic organic small molecules into existing matrices or sunscreen formulations [9,10]. Such organic small molecules generally possess conjugated structures or photo-responsive chemical bonds. Upon UV excitation, these molecules can transition from the ground state to excited states and dissipate energy as heat through nonradiative pathways, such as vibrational relaxation and internal conversion, or consume energy via molecular photoisomerization [11]. However, these organic small molecules suffer from insufficient photostability; their incorporation into sunscreens and other products can readily induce pronounced transdermal effects and may also pose risks to ecosystems. By contrast, the UV-protective function of inorganic materials is primarily provided by inorganic nanoparticles [12,13], such as ZnO and TiO_2_, which achieve physical UV shielding mainly through reflection and scattering [14]. Nevertheless, to achieve satisfactory UV protection, the relatively high loading required for such inorganic nanomaterials can compromise the transparency and mechanical properties of film materials, the skin-feel experience of sunscreen products, and the environmental biosafety of the materials. Recently, the development of novel materials with intrinsic UV-protective functionality has been a research hotspot in polymer chemistry and materials science. Some progress has been made in recent years in the preparation of new UV-protective polymers using efficient polymer synthesis methods [15,16,17].

Multicomponent reactions (MCRs) are classic organic reactions that date back to the synthesis of amino acids by Strecker in 1850 [18,19]. MCRs combine three or more reactants into a single product in one pot under mild conditions [20,21]. Since Meier et al. successfully synthesized polymers via Passerini reaction [22], more and more MCRs, such as the Ugi reaction [23,24,25], Biginelli reaction [26,27], Hantzsch reaction [28,29,30], Kabachnik–Fields reaction [31,32,33], and Debus–Radziszewski reaction [34,35,36], as well as metal-catalyzed and alkyne-based MCRs [37,38,39], have been applied in the development of new polymers. MCRs are highly efficient and atom-economical, so they are well-suited to the development of intrinsic UV-protective polymers. Four factors account for the UV-protective function of MCR-based polymers. (1) Some MCR products contain heterocycles that absorb UV rays, such as the 1,4-dihydropyridine (1,4-DHP) moiety from the Hantzsch reaction and the 1,4-dihydropyrimidin-2(1H)-thione (DHPMT) scaffold from the Biginelli reaction. (2) MCRs accept a wide range of reactants, so natural aldehydes and amines with strong UV absorption can be readily incorporated into the polymers. (3) MCR-based polymers sometimes possess anti-inflammatory and antioxidant activities. They quench the reactive oxygen species (ROS) generated by UV radiation and thus reduce UV-induced damage. (4) MCRs are compatible with polymerizations such as radical polymerization and condensation polymerization, so the UV-protective groups can be intactly included in the resulting polymers.

**Scheme 1:**
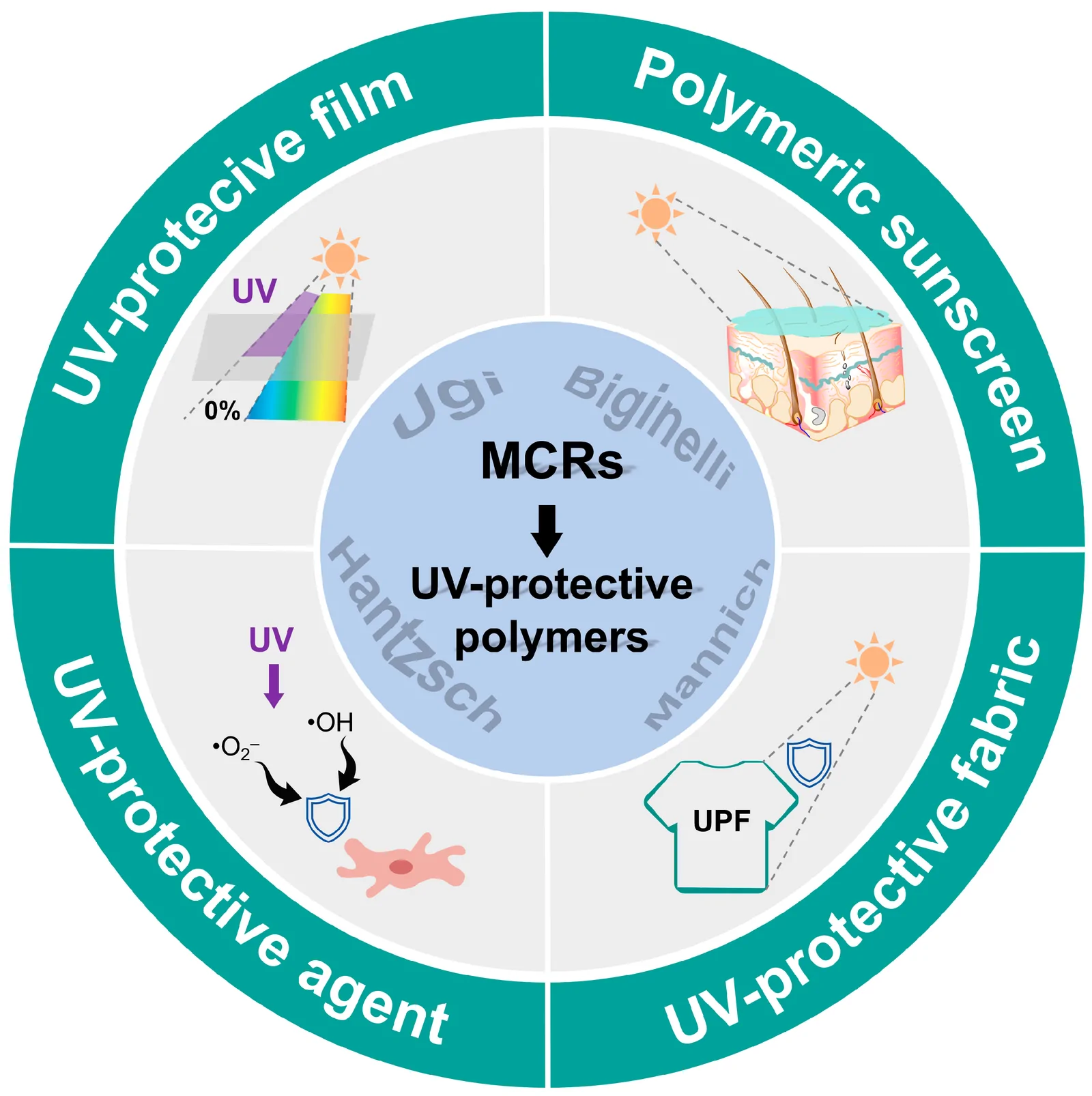
UV-protective polymers developed by multicomponent reactions.

We note that most existing reviews focus on inorganic/organic UV-blocking additives for the development of UV-protective polymers, whereas reviews built around intrinsic UV-resistant polymers remain scarce, which is one of the reasons why materials development still tends to rely on reformulating existing additive systems. In recent years, the design and synthesis of intrinsic UV-protective polymers through MCRs have been reported in numerous leading journals, and several distinctive molecular design strategies, together with their practical UV-blocking performance, have attracted considerable interest. A timely summary of progress in developing UV-resistant polymers by MCRs would therefore benefit researchers both in further improving material performance and in moving these materials toward practical large-scale production. In this mini-review, we will focus on the UV-protective polymers developed by MCRs, including UV-protective agents for cells, polymeric sunscreen, multifunctional UV-protective film, and UV-protective fabric (Scheme 1). We selected articles from Web of Science (all databases) by combining “multicomponent reaction(s)” and “MCR” with “UV-resistant”, “UV-protective”, “UV-blocking”, “UV filter”, and “sunscreen” with preference for the most recent work.

We will introduce the design concepts and synthesis methods of these polymers, evaluate their UV resistance, and also examine the anti-UV performance that MCRs bring to the polymers (Table 1). We believe that presenting these recent advances can provide guidance at the level of molecular structure design for the development of intrinsic UV-resistant materials. We will also briefly introduce some recent advancements in the research of polymers for protecting against radiation across a wider range of wavelengths. Finally, we will discuss the future UV-protective polymers developed by MCRs and the possibility of introducing other valuable functions to broaden their application.

## 2. Development of UV-Protective Polymers

### 2.1. UV-Protective Agent for Cells

UV-induced cell damage is a complex, multistage and multi-target process. First, UV radiation can generate various ROS, within cells or in the surrounding environment, including superoxide anions (•O_2_^–^) and hydroxyl radicals (•OH), which attack biomacromolecules, disrupt cell membrane integrity, inactivate enzymes, and cause damage to intracellular lipids, proteins, and DNA [51,52,53]. Second, UV radiation damages intracellular DNA directly, which interferes with DNA replication and transcription and can ultimately cause disease [54]. UV-protective materials for biological use must therefore be tested for UV protection, ROS scavenging, and biosafety at the cellular level. However, some organic small-molecule agents and inorganic nanomaterials disperse or dissolve poorly in cell culture media. In contrast, UV-protective monomers developed through MCRs can be copolymerized with water-soluble monomers, and the resulting UV-protective polymers generally exhibit favorable biosafety and water solubility. Macromolecular agents also possess high, safe concentrations, and disperse or dissolve well; thus, they outperform small molecules in cell-level assays [55].

**Figure 1:**
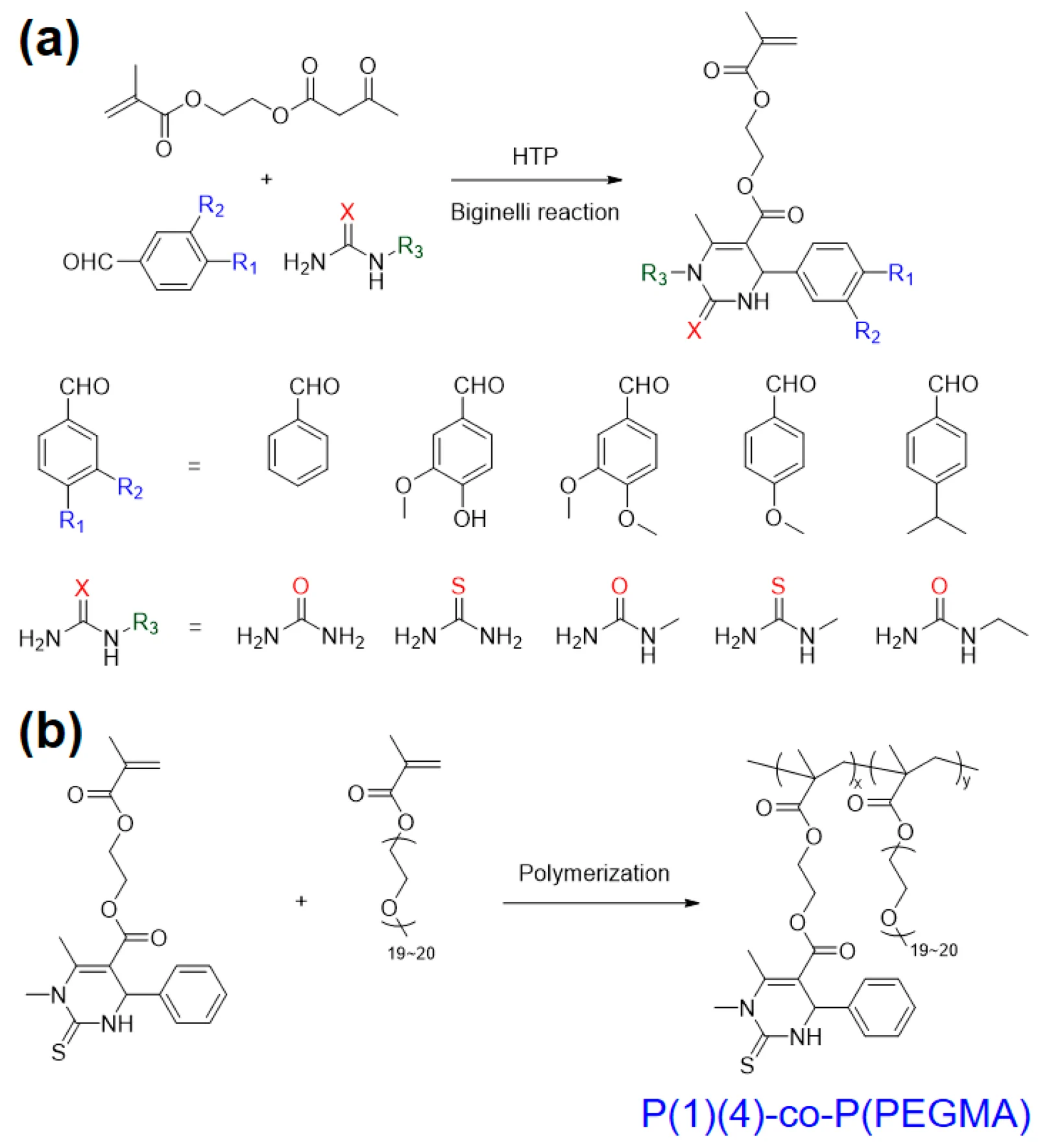
High-throughput synthesis of UV-protective agents for cells through the Biginelli reaction. (**a**) High-throughput synthesis of 25 monomers using aldehyde extracts from natural essential oils. (**b**) Synthetic route to the representative water-soluble polymer P(1)(4)-co-P(PEGMA).

In 2018, Tao et al. prepared 25 functional monomers through a high-throughput (HTP) system based on the Biginelli reaction, using natural small-molecule aldehyde compounds extracted from essential oils, urea, or thiourea derivatives, and β-ketoester monomers [40] (Figure 1a). These monomers are amenable to radical polymerization with the water-soluble monomer poly(ethylene glycol) methyl ether methacrylate (PEGMA), affording water-soluble polymers (Figure 1b). From the results of the ^1^H nuclear magnetic resonance (NMR), the polymers retained the functional groups on the corresponding monomers, confirming the compatibility of the Biginelli products with free radical polymerization. Subsequently, the resulting Biginelli-based water-soluble polymers were evaluated by cell-protection experiments under UV irradiation (i.e., 15 min of UV exposure followed by 24 h of co-culture in polymer-containing medium), which led to the identification of polymer P(1)(4)-co-P(PEGMA) as the candidate with the optimal UV-protective capacity. In the cell fluorescein diacetate/propidium iodide (FDA/PI) double-staining experiment, the cells protected by P(1)(4)-co-P(PEGMA) almost all survived, while the cells protected by the superoxide dismutase (SOD) almost all died. The results indicated that the P(1)(4)-co-P(PEGMA) effectively scavenged ROS generated under UV irradiation, prevented oxidative stress, and thereby protected cells, with efficacy even superior to that of SOD. This study highlights the advantages of MCRs in introducing functional groups into polymers, developing macromolecular therapeutics, and facilitating screening.

**Figure 2:**
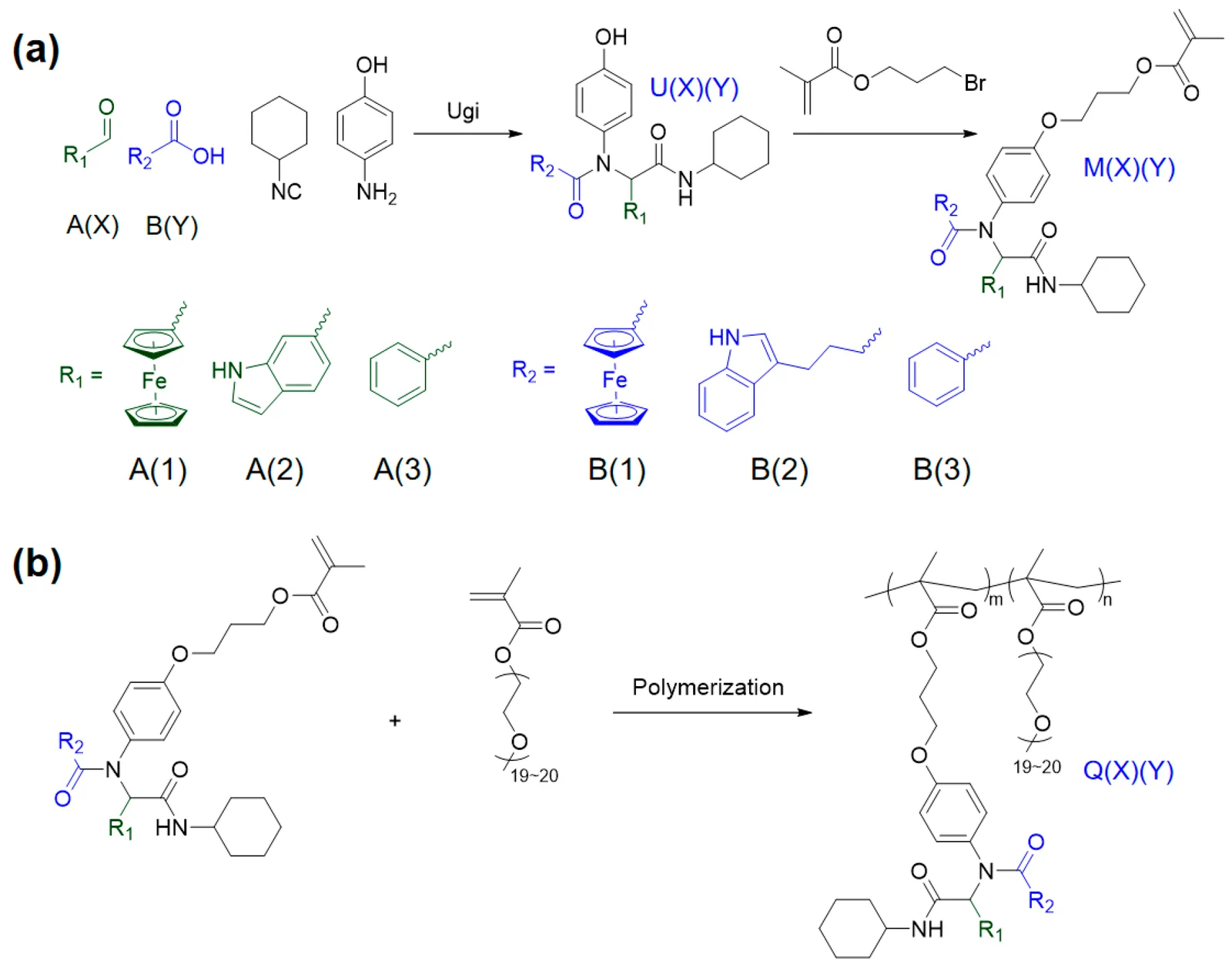
High-throughput synthesis of UV-protective agents for cells through the Ugi reaction. (**a**) High-throughput synthesis of Ugi monomer. (**b**) Synthetic route to water-soluble polymers through radical polymerization.

To expand the use of other MCRs in HTP synthesis and UV-protective screening, and to reduce the cell oxidative stress damage caused by UV radiation, the same group developed a series of antioxidant monomers and polymers through the four-component Ugi reaction [41] (Figure 2a,b). In this work, they obtained different Ugi products (U(X)(Y)) by adjusting the groups on the aldehyde and carboxylic acid reactants. These products were converted into functional monomers (M(X)(Y)), and the monomers were copolymerized with PEGMA to give the antioxidant polymers (Q(X)(Y)). Electron paramagnetic resonance (EPR) showed that Q(1)(1), Q(1)(2) and Q(1)(3) scavenge ROS efficiently. Then, they verified the ability of the selected polymers to protect DNA under UV radiation. Agarose gel electrophoresis of calf thymus deoxyribonucleic acid sodium salt (CT-DNA) showed that all the polymers protect DNA, and Q(1)(1) was the most effective against UV-induced oxidative damage.

### 2.2. Polymeric Sunscreen

UV radiation in sunlight not only causes acute skin damage, such as erythema and sunburn, but may also accelerate cutaneous photoaging, including wrinkle formation, and can even induce immunosuppression and skin carcinogenesis. Applying sunscreen to the skin is a convenient and simple option for daily protection against UV radiation. However, the extensive use of sunscreens may have catastrophic effects on marine ecosystems. For example, the massive release of 2-hydroxy-4-methoxybenzophenone (oxybenzone), an organic UV filter in sunscreens, into the ocean has led to coral bleaching [56,57]. Similarly, inorganic UV filters such as ZnO nanoparticles can also enter the environment and inhibit plant growth [58]. Therefore, the development of eco-friendly water-soluble sunscreens would facilitate the achievement of sustainable development goals.

**Figure 3:**
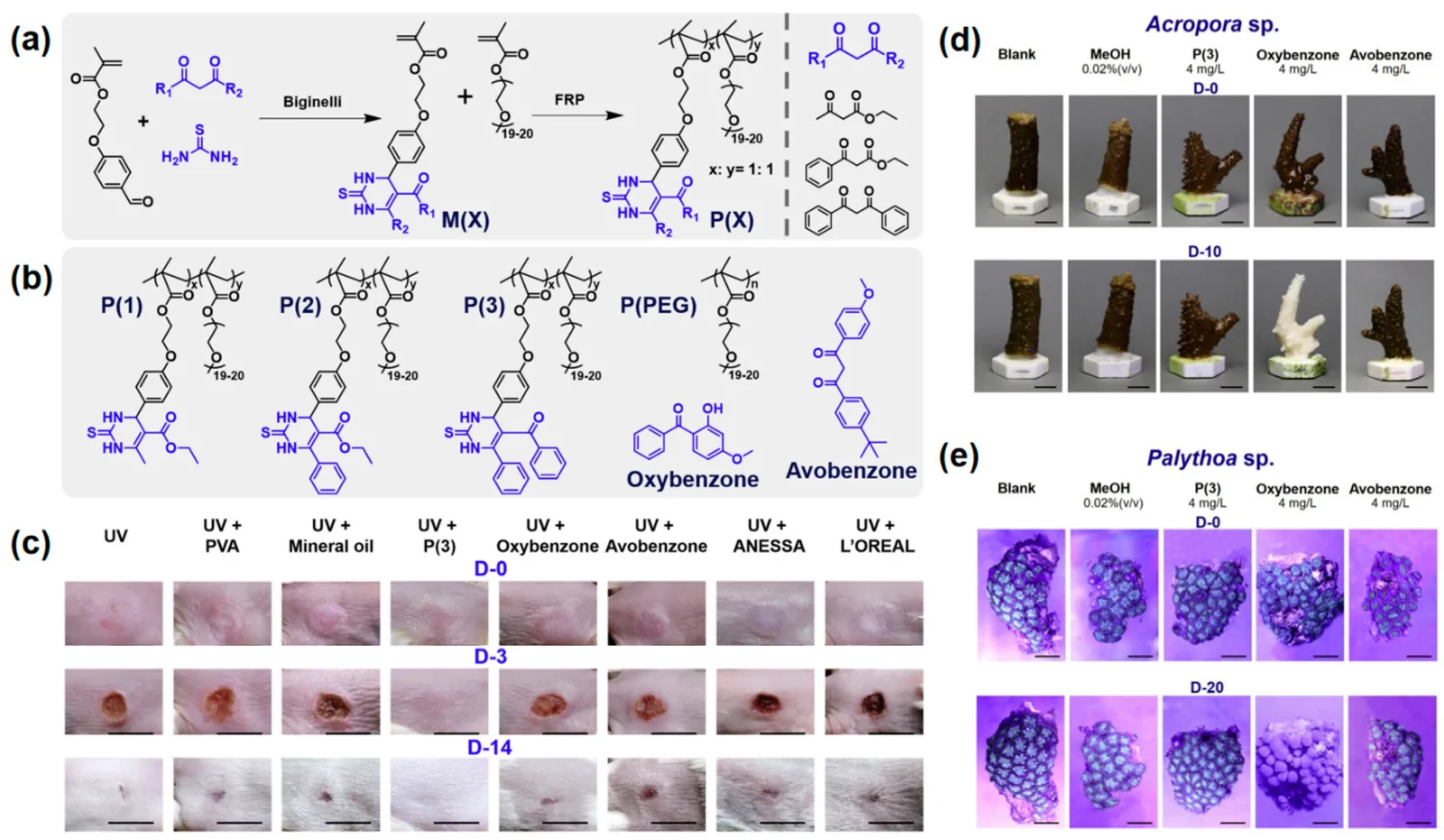
Coral-friendly polymeric sunscreens. (**a**) Route to water-soluble polymers through the Biginelli reaction and radical polymerization. (**b**) Chemical structures of the polymers and small molecules used for the evaluation of UV resistance and biosafety. (**c**) Protective performance of the polymeric sunscreen compared with the blank and commercial sunscreens. (**d**) Bleaching effect of several UV-protective agents on Acropora sp. (**e**) Toxicity of several UV-protective agents toward Palythoa sp. Reprinted with permission from ref. [42].

Tao et al. prepared a series of water-soluble UV-resistant polymers through the Biginelli reaction [42] (Figure 3a). In this study, they prepared monomers with different functional groups through the Biginelli reaction, and copolymerized these monomers with PEGMA to produce water-soluble polymers P(X) (Figure 3b), among which P(3) gave the best UV protection and was selected for testing on mouse skin. Under strong UV radiation, P(3) could protect the mice from UV-induced skin burn. The protection effect of P(3) is better than those of the organic molecules (oxybenzone and avobenzone) and the commercial sunscreens (ANESSA and L’OREAL) (Figure 3c).

Moreover, compared with oxybenzone, P(3) caused almost no damage to corals, so it performs well in terms of ecological friendliness (Figure 3d,e). Additionally, the high molecular weight of P(3) also prevents transdermal absorption, which ensures its biosafety.

**Figure 4:**
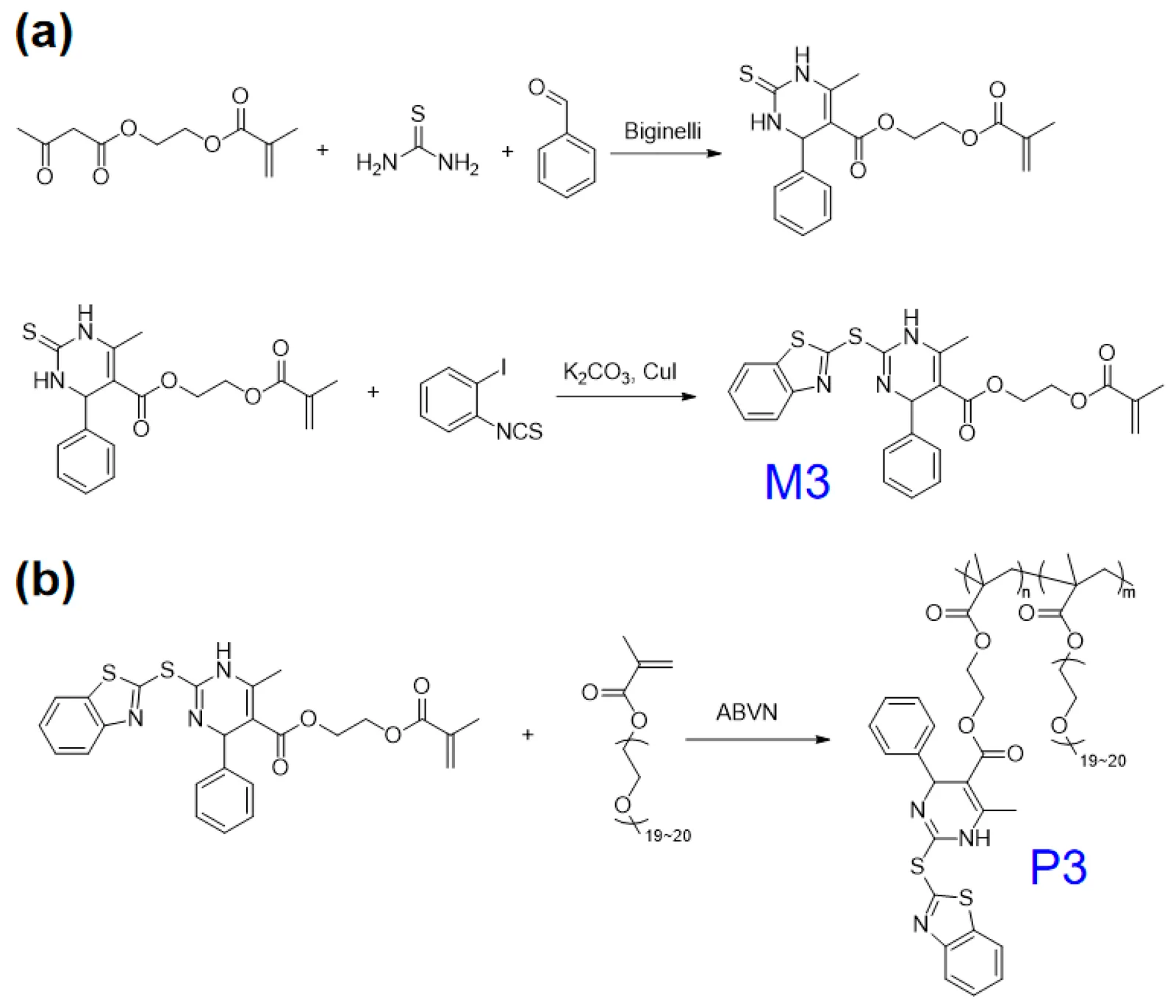
The UV resistance of polymers can be further enhanced by post-modification of functional groups. (**a**) Post-modification of DHPMT groups, which couples UV-protective groups onto the original chemical structure of the monomer. (**b**) Synthetic route to water-soluble polymer P3 through the radical polymerization.

Mao and Dong further utilized the DHPMT heterocyclic ring of the Biginelli monomer for further post-modification [43] (Figure 4a). They coupled a series of aromatic groups at the sulfur site of the Biginelli monomers, and then copolymerized the monomers with PEGMA to prepare a series of water-soluble polymers (Figure 4b). The cell safety and UV protection of these polymers were evaluated at the cellular level, and the polymer with benzothiazole groups was selected to prepare polymeric sunscreen S3. The sunscreen showed superior performance in vivo to the commercially available sunscreen ANESSA in protecting the skin against both UVA and UVB (Figure 4b). This work shows that post-modification can further improve the UV protection of Biginelli-based products, highlighting the value of MCRs in molecular structure design to achieve new functional polymers.

**Figure 5:**
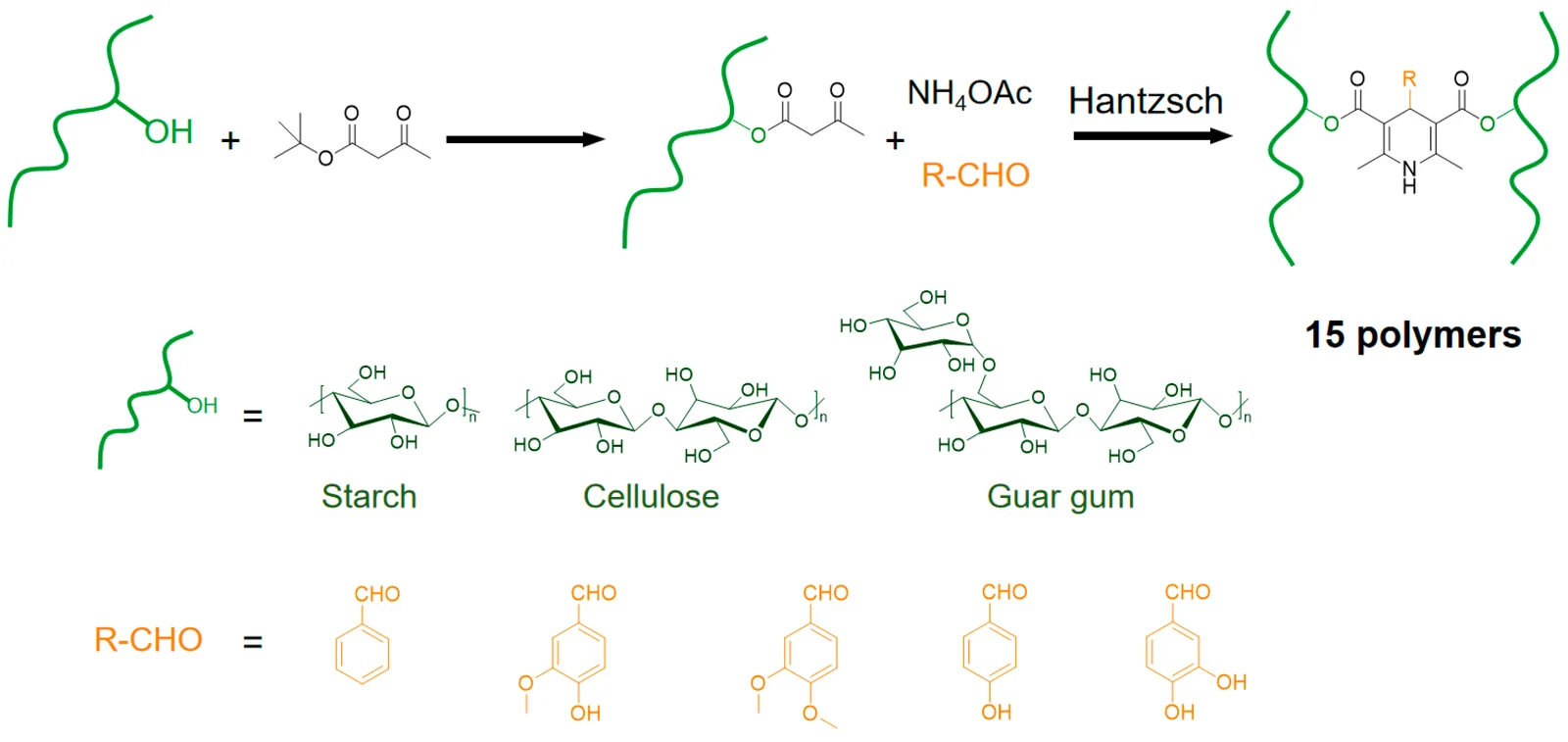
Preparation of polymeric sunscreens by modification of natural macromolecules.

Modifying existing natural polymers for UV protection also preserves their intrinsic biodegradability and biocompatibility. Qi et al. grafted β-ketoester structures onto natural polymers such as starch, cellulose, and guar gum, and then post-modified them with aldehyde extracts from essential oils through the Hantzsch reaction to construct UV-protective and antioxidant biopolymers [44] (Figure 5). Taking cellulose as a representative reactant, the ^1^H NMR spectra confirmed that the post-modification took place and the functional groups were introduced successfully. The polymer with the best solubility, R(2)(5), was selected for the preparation of the sunscreen. This sunscreen could shield UV radiation directly and also efficiently scavenged the free radicals generated by UV radiation. The radical quenching experiment and UV transmittance results indicated that the radical scavenging and the UV-shielding capacity of sunscreens could be both improved as the R(2)(5) content increased.

**Figure 6:**
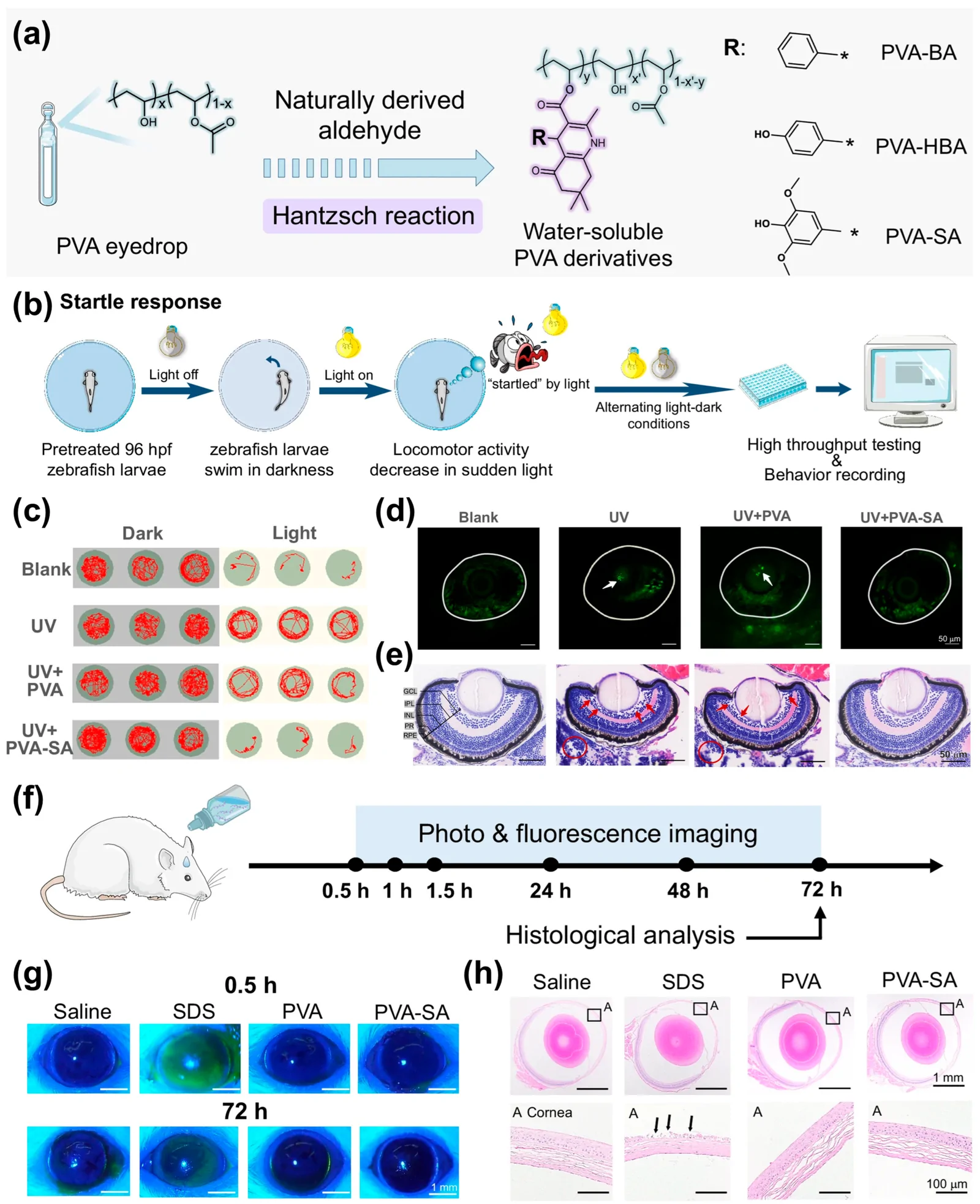
Preparation of UV-protective eye drops through the Hantzsch reaction. (**a**) Preparation of water-soluble polymers by modification of PVA. (**b**) Startle response experiment on zebrafish under alternating light and dark conditions. (**c**) Moving trajectories of the zebrafish in the startle test. The trajectories of the polymer-protected zebrafish were similar to those of zebrafish not exposed to UV irradiation. (**d**) Cell apoptosis in the eye observed by acridine orange fluorescence staining. The eyes of the polymer-protected zebrafish were similar to those of zebrafish not exposed to UV irradiation, whereas apoptosis occurred in the unprotected and PVA-only groups. (**e**) H&E staining of zebrafish eye sections. The eyes of the polymer-protected zebrafish were similar to those of zebrafish not exposed to UV irradiation, whereas the unprotected and PVA-only groups showed clearly inflammatory cell infiltration. (**f**) Scheme of experimental procedures of the eye irritation test. (**g**) Representative slit-lamp images of fluorescein cornea staining of mouse eyes after different treatments, after 0.5 and 72 h. Scale bar: 1 mm. (**h**) H&E staining of mouse eyes after different treatments for 72 h. Scale bar: 1 mm. The bottom row shows enlarged images of the cornea. The arrows indicate the ruptured cells of the cornea in the sodium dodecyl sulfate (SDS) group. Scale bar: 100 μm. Reprinted with permission from ref. [45].

Beyond conventional skin protection, UV protection of the eyes has likewise attracted the interest of researchers. Excessive UV irradiation can induce a variety of ocular diseases, such as pterygium, cataract and photokeratitis [59,60]. Common approaches to ocular UV protection, such as wearing sunglasses, usually offer incomplete protection caused by back reflection that is difficult to eliminate, or from the fact that infants and young children find them difficult to wear and thus remain unprotected [61]. To address this problem, Tao et al. developed an eye sunscreen that is safe, highly transparent, and water-soluble, and can be used directly as eye drops [45]. They selected poly(vinyl alcohol) (PVA), the polymeric component of artificial tears, as the basis, and modified it with benzaldehyde, p-hydroxybenzaldehyde, and syringaldehyde groups through diketene modification and the Hantzsch reaction, affording three different polymers—PVA-BA, PVA-HBA, and PVA-SA. The resulting polymers all exhibited favorable UV absorption capacity in aqueous solutions, among which PVA-SA exhibited the best UV resistance (Figure 6a). They evaluated the effectiveness of the polymer in protecting against UV radiation by observing the changes in the eye vision of zebrafish after exposure to UV light. The zebrafish with normal and abnormal eye vision exhibited different responses to the alternating light and dark environment (Figure 6b). According to the statistical results, in the light environment, zebrafish protected by PVA-SA exhibited a startle response similar to that of normal zebrafish, whereas unprotected zebrafish or zebrafish treated only with a PVA solution showed a slow response, indicating that the eye sunscreen helps to protect the vision of zebrafish (Figure 6c). The results of fluorescence staining and H&E staining of zebrafish eye sections also confirmed that the eyes of unprotected or PVA-protected zebrafish exhibited significantly apoptotic cells and inflammatory cell infiltration around the eyes, while the eyes protected by PVA-SA showed almost no difference from those of normal fish (Figure 6d,e). It is also worth mentioning that this polymeric eye sunscreen has a relatively long retention time, conducive to its long efficacy. The eye irritation test (Figure 6f) showed that the fluorescein sodium staining of the cornea at 0.5 h after instillation, together with the H&E staining at 72 h, revealed no significant difference in ocular irritancy between PVA-SA and PVA, the main component of artificial tears (Figure 6g,h). This indicates that the eye sunscreen causes nearly no obvious acute irritation.

### 2.3. Multifunctional UV-Protective Film

Prolonged UV exposure weakens the mechanical properties of polymeric materials, causing them to become brittle or have cracks. This degradation results from the combined effects of UV-induced radical reactions, oxidation reactions, and polymer chain scission. Radical attack on the polymer chains also creates carbonyl, hydroxyl, and carboxyl groups. These groups act as new chromophores and deepen the color of materials [62]. Meanwhile, textiles, automotive coatings and artworks fade under prolonged UV irradiation. Dyes absorb light energy and then undergo oxidation, reduction, and isomerization, which alter or destroy their chromophore structures [63]. Therefore, to mitigate UV-induced changes in material properties, the development of UV-protective film materials is essential. MCRs can provide new strategies for the molecular design of high-performance UV-protective films.

**Figure 7:**
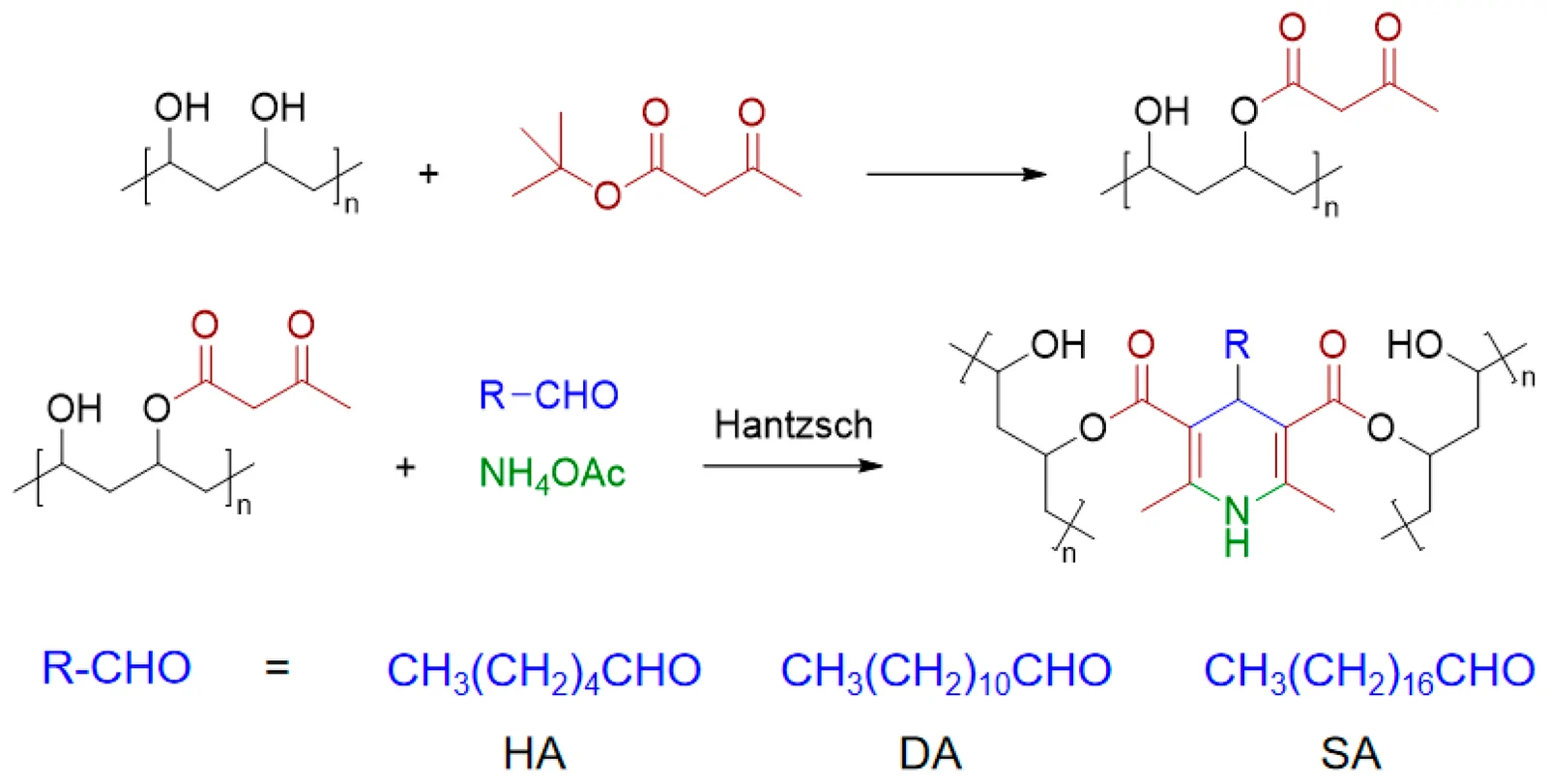
Hydrophobic and UV-resistant modification of PVA-based films.

Modifying commercially available synthetic polymers on the surface is a convenient way to prepare UV-blocking films. Qi et al. prepared a PVA-based UV-blocking film via surface modification [46] (Figure 7). They used transesterification to introduce β-ketoester groups into PVA, which gave poly(vinyl alcohol) acetoacetate (PVAA). After casting the PVAA into a film, it was surface-modified at room temperature by the Hantzsch reaction in a solution containing NH_4_OAc and hydrophobic aldehydes. Elemental mapping by scanning electron microscopy (SEM) showed that the surface modification was successful and uniform. The contact angle test confirmed the hydrophobic modification of PVA. Due to the introduction of DHP groups, the PVA-based films (HA-PVA, DA-PVA and SA-PVA) had excellent UV-blocking ability in the UVA (315–400 nm) and UVB (280–315 nm) wavelength ranges, and also had good visible light transmittance. Importantly, the surface modification had almost no significant influence on the mechanical properties of the film, which could still be bent and knotted and could lift a load of about 5 kg. Moreover, after being exposed to UV radiation (365 nm, 20 W/m^2^) for 500 h, the UV-blocking properties of these films remained almost unchanged. This work shows the value of MCRs in simple surface modification. However, the chemical structure and mechanical strength of the films may change after 500 h of irradiation, and this aspect deserves further investigation.

**Figure 8:**
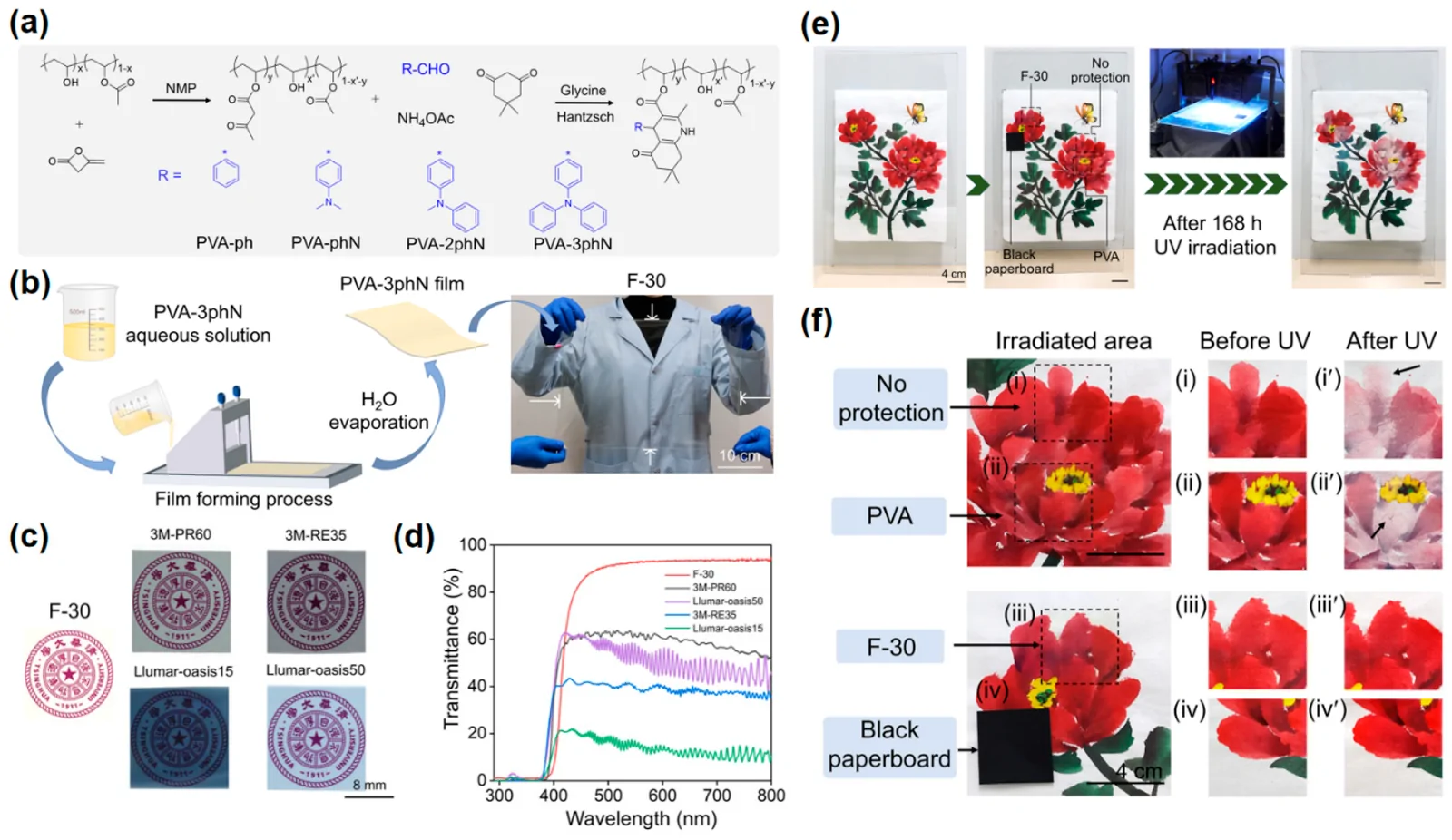
Preparation of polymer films through the Hantzsch reaction for the protection of artworks. (**a**) Synthetic route to the polymers. (**b**) Blade-coating and preparation of the F-30 film. (**c**) Transparency of F-30 and of commercial UV-protective films. (**d**) UV absorption of F-30 compared with other commercial UV-protective films. (**e**) UV irradiation of a Chinese painting and its protection by various materials. (**f**) Magnified details of the Chinese painting under protection by the various materials. (**i**) and (**i′**) No protection; (**ii**) and (**ii′**)PVA; (**iii**) and (**iii′**) F-30; (**iv**) and (**iv′**) Black paperboard. Reprinted with permission from ref. [47].

Homogeneous post-modification is another route to the creation of UV-protective films. Tao et al. modified PVA dissolved in N-methylpyrrolidone (NMP) with diketene, and then post-modified the resulting PVA-ketoester with 5,5-dimethyl-1,3-cyclohexanedione and a series of aromatic aldehydes to obtain several polymers via the Hantzsch reaction [47] (Figure 8a). PVA-3phN, which carries three phenyl groups, absorbed UV light most strongly among all samples. It was dissolved in water and blade-coated into a film (F-30, 30 µm thick) (Figure 8b), which is superior to commercial UV-protective films in both UV-blocking ability and transparency (Figure 8c,d). They then used F-30 to protect a Chinese painting (Figure 8e). After 168 h of UV irradiation, the color of the areas protected by F-30 was almost unchanged, comparable to that of the areas shielded with black cardboard. The areas protected only by a conventional PVA film, or left unprotected, faded markedly (Figure 8f).

### 2.4. UV-Protective Fabric

Among many methods of shielding against UV radiation, wearing suitable UV-protective fabrics is a convenient option. Multifunctional fabrics based on traditional natural polymers are highly compatible with human skin and readily biodegradable in the environment, which is consistent with the goals of sustainable development; consequently, they are highly favored across a wide range of application scenarios [64]. Unlike the requirements for sunscreen and films, fabrics usually need to maintain their UV resistance without significant changes after multiple washes. MCRs, characterized by high reaction efficiency and mild reaction conditions, allow the functional groups of natural polymers to be exploited efficiently for functional modification without disrupting the polymer backbone, thereby endowing these materials with a variety of different functions. Moreover, surface functionalization through MCRs forms stable covalent bonds directly on the fabric, anchoring the functional groups to the surface and thereby minimizing the possibility of performance loss during repeated washing. Such anchored groups are retained better than coating layers that may be removed by surfactants, repeated washing, and mechanical friction.

**Figure 9:**
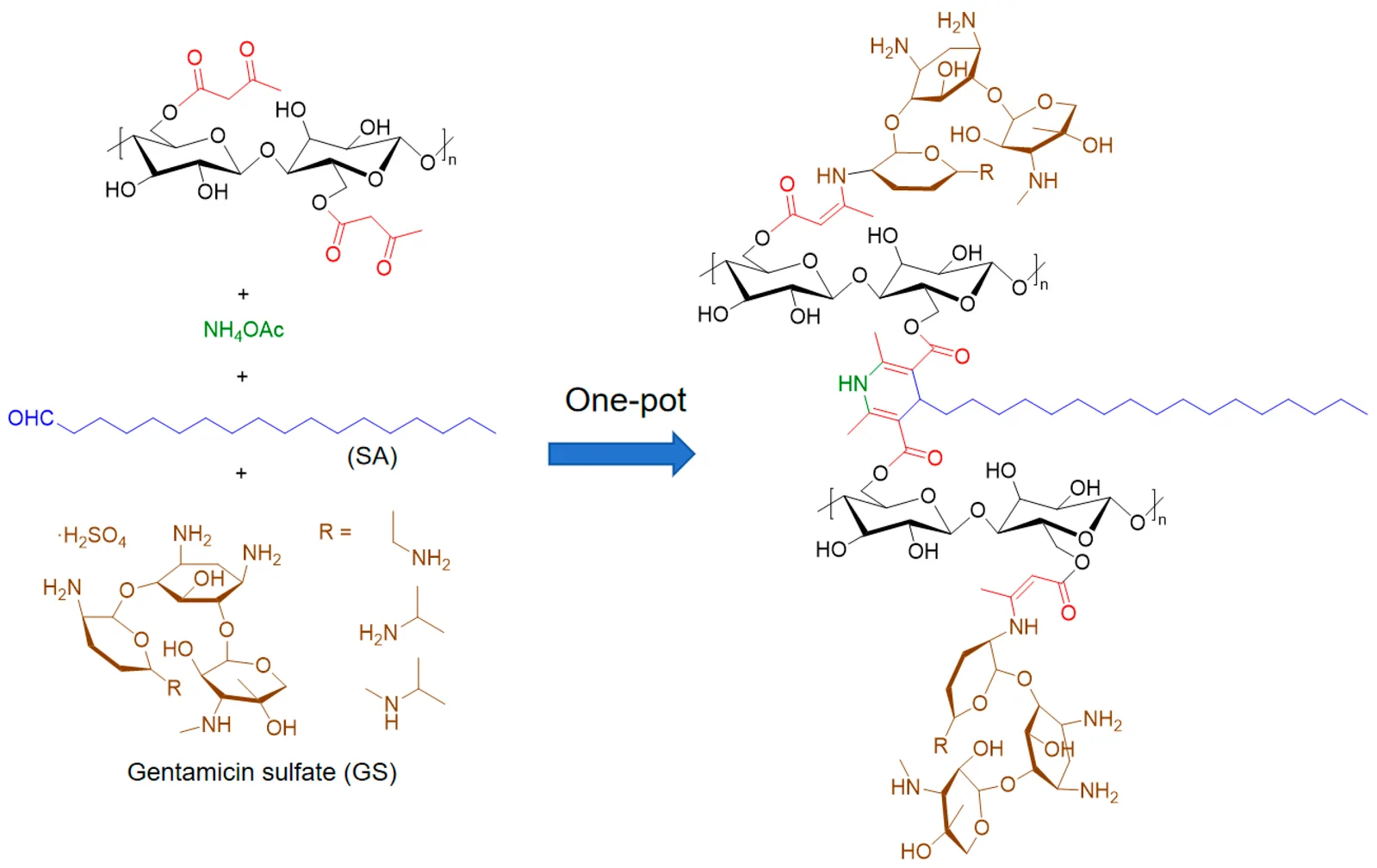
UV-resistant, hydrophobic, and antibacterial surface modification of existing cotton fabrics through the Hantzsch reaction.

Multifunctional cotton fabrics are widely used, but their functional modification and scalable production remain challenging. Qi et al. introduced β-ketoester groups onto cotton fabrics by mild surface modification. The β-ketoester groups could react with stearaldehyde and NH_4_OAc through the Hantzsch reaction, which grafted hydrophobic alkyl chains and 1,4-DHP structures onto the fabric surface. The modified fabric was more hydrophobic than the pristine cotton and also showed better UV resistance [48] (Figure 9). In addition, the β-ketoester groups could also react with gentamicin sulfate to immobilize the antibiotic directly on the fabric surface, endowing the fabric with antibacterial activity. The modified fabric kept its original white color, and fluorescence imaging showed that the modification proceeded uniformly across the fabric. It also shielded UV light more strongly than unmodified cotton fabric, and its ultraviolet protection factor (UPF) remained almost unchanged after multiple washing cycles. In addition, the modification did not sacrifice the mechanical integrity or the breathability of the fabric: the tensile strength remained close to that of pristine cotton, the breaking elongation increased slightly, and the air permeability even improved. This research indicates that MCRs can be used as highly efficient tools for heterogeneous surface modification, and that the existing fabric materials can be given several additional functions through the surface modification.

**Figure 10:**
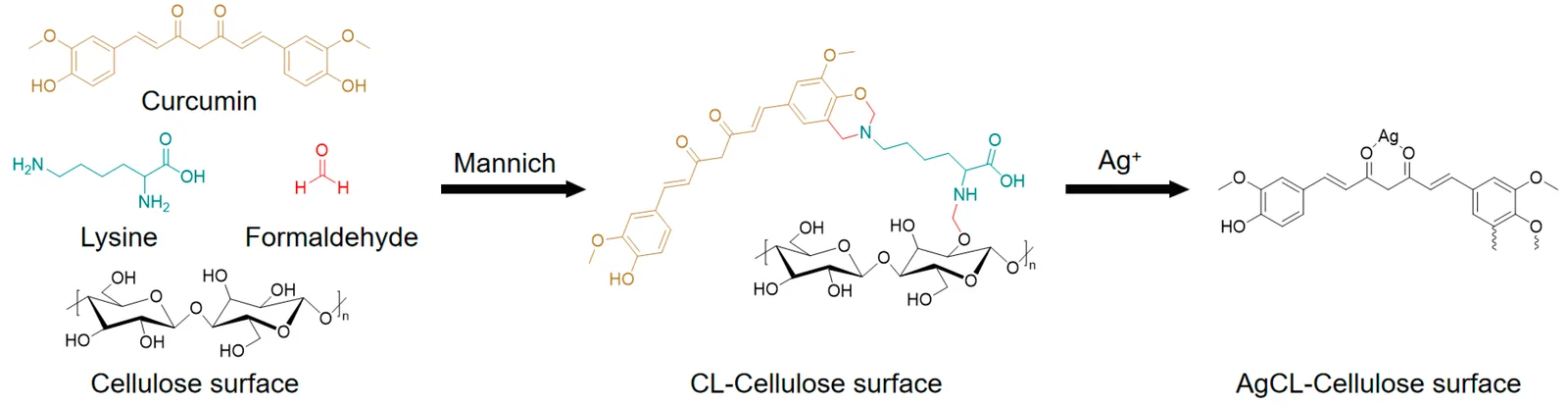
UV-resistant and antibacterial surface modification of existing fabrics through the Mannich reaction.

In a further study, Qi et al. post-modified sulfated cellulose nanofibers (SCNF) via the Mannich reaction, grafting the natural products curcumin and lysine onto SCNF, with the grafted curcumin moieties serving to chelate and disperse silver particles (AgCL-SCNF) [49] (Figure 10). The AgCL-SCNF system was dissolved in the solvent and sprayed directly onto masks, which gave the materials UV-protective and antibacterial properties. Furthermore, they attempted the large-scale preparation of functional materials directly on cotton fabric. The modified cotton fabric also shielded UV radiation well, and its appearance changed little after several washing cycles.

## 3. Extension: Protection Against Other Harmful Radiations

### 3.1. Dynamic Covalent Polymer Films with UV-Protective Functions

**Figure 11:**
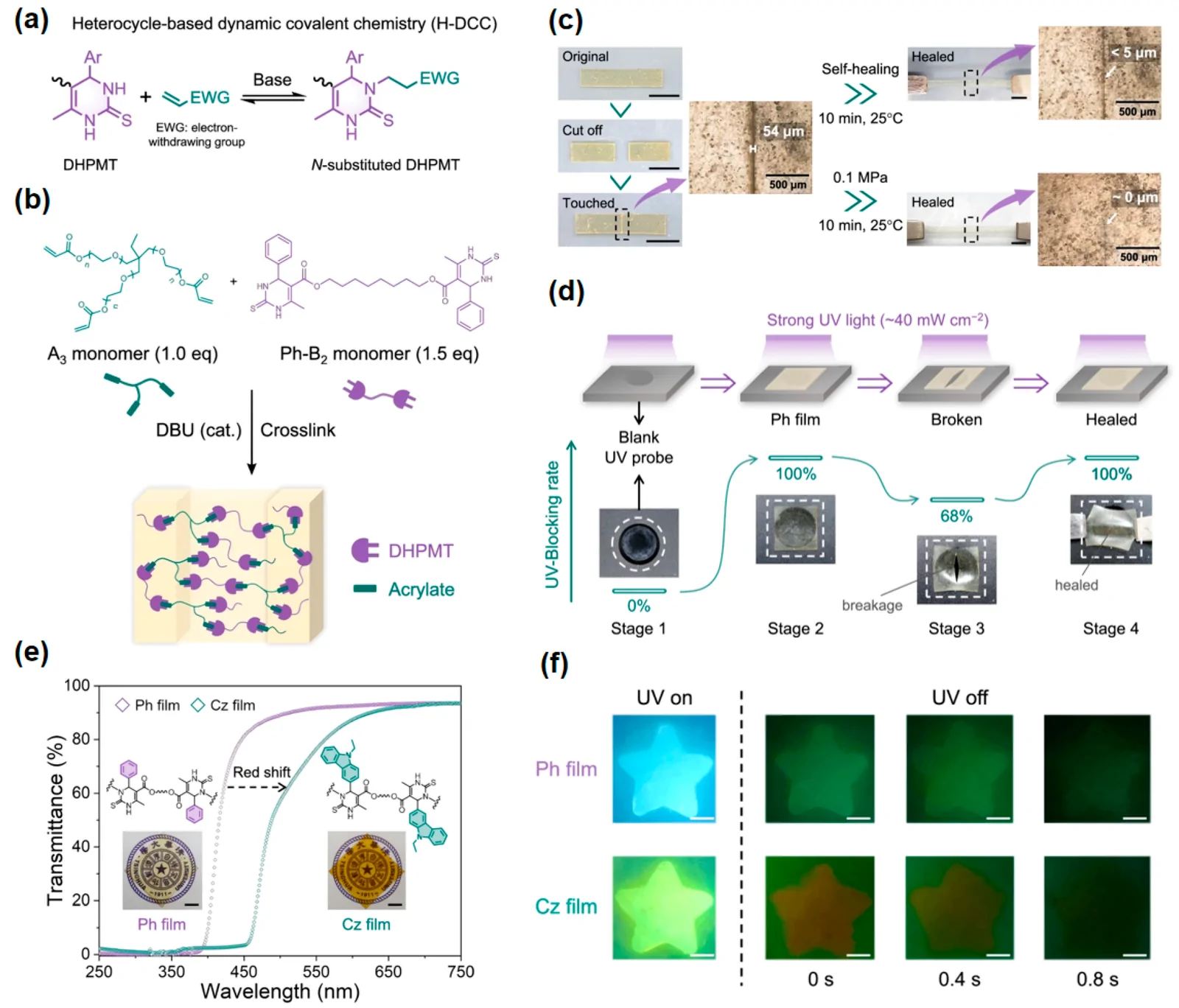
Dynamic film materials with additional UV-protective functions. (**a**) Dynamic chemistry based on DHPMT. (**b**) Construction of the dynamic materials. (**c**) Self-healing properties of the materials. (**d**) Self-healing of the damaged materials, and the recovered ability to shield UV light. (**e**) Absorption wavelength of the dynamic materials tuned by changing the aldehyde substrates. (**f**) Long afterglow of the materials obtained by changing the aldehyde substrates. Reprinted with permission from ref. [50].

Combining UV protection with dynamic covalent chemistry is a new research direction. Tao et al. studied the reversibility of the Michael addition between the DHPMT heterocycle and electron-deficient alkenes (Figure 11a). They crosslinked the phenyl-substituted 2-DHPMT monomer (Ph-B2) with the three-arm acrylate monomer (A3) under the catalysis of 1,8-diazabicyclo[5.4.0]undec-7-ene (DBU), thereby preparing dynamic materials [50] (Figure 11b). Owing to the low activation energy of this dynamic chemical reaction, the films made from this material can undergo self-healing after cutting at room temperature (Figure 11c). The repaired films not only regain their strength but also restore their excellent UV resistance, thereby extending their application lifespan (Figure 11d). In addition, by selecting appropriate aldehyde reactants, the absorption wavelength of the film could have a red shift (Figure 11e) and exhibit long afterglow properties (Figure 11f). This work demonstrates a new strategy for expanding the functionality of MCR-based anti-UV polymers.

### 3.2. Protection Against Harmful Blue Rays

**Figure 12:**
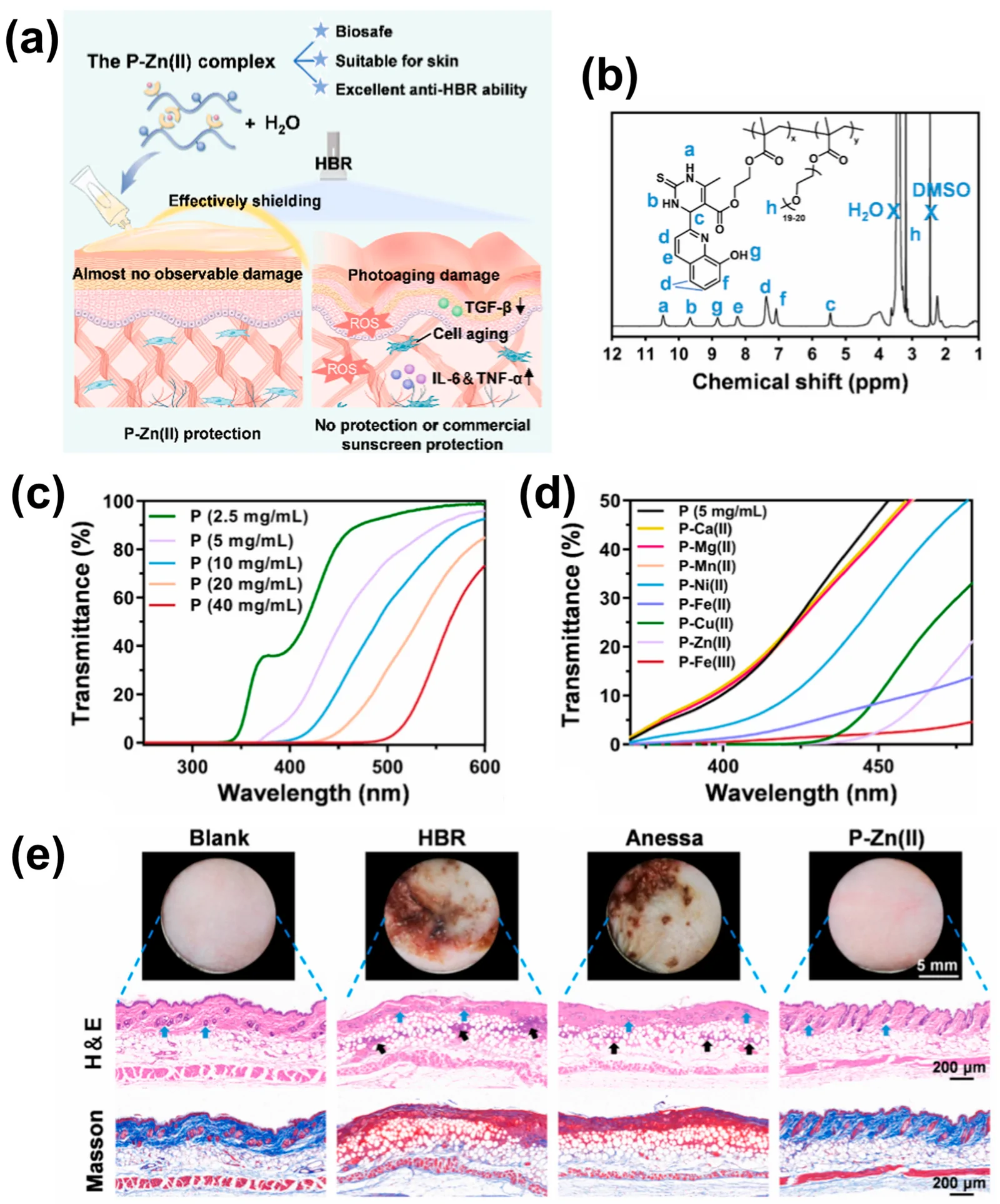
Protection against harmful blue rays. (**a**) Development of a polymer-Zn(II) (P-Zn(II)) complex against harmful blue rays via the Biginelli reaction. (**b**) Chemical structures of the polymer and its ^1^H NMR spectrum. (**c**) UV–vis absorption spectra of the polymers. (**d**) Red shift in the UV absorption after chelating zinc ion. (**e**) Optical photographs, H&E staining, and Masson staining of the skin under different treatments. Reprinted with permission from ref. [65].

Harmful blue rays (HBR, 400–450 nm) from the sun and many electronic devices can induce skin photoaging. HBR exposure in daily life is far from negligible: a cumulative HBR dose of 20 J/cm^2^, which clearly damages skin cells, corresponds to only about 4.5 h of sunlight and to about 80 days of mobile-phone use, 118 days of computer use or 7 days of indoor LED lighting. However, common sunscreens have poor HBR-blocking ability, while traditional anti-HBR materials are unavailable for skin application. Tao et al. also used the Biginelli reaction to introduce metal ions and coordinating groups into the polymer structure, thereby achieving a polymer with HBR-resistance (Figure 12a) [65]. A functional monomer with 8-hydroxyquinoline structure was prepared via the Biginelli reaction and copolymerized with PEGMA to afford a water-soluble polymer (Figure 12b). The polymer could chelate zinc ions (Zn(II)) by the hydroxyquinoline groups to form the complex P-Zn(II), achieving an overall red shift in the UV–vis absorption (Figure 12c,d). When formulated into a blue-light protective agent, the P-Zn(II) complex effectively prevented skin damage caused by prolonged blue-light irradiation and protected tissues from blue-light-induced oxidative stress, senescence, and death (Figure 12e). Such a molecular design strategy can provide a useful reference for the design of new blue-light-protective lenses or screen-protective films for electronic devices.

## 4. Perspectives and Conclusions

As concerns over the effects of UV radiation on human health, the durability of materials, and the environment continue to grow, the development of efficient, stable, safe, and environmentally friendly UV-protective materials has become an important research direction in the field of functional polymers. In this mini-review, we provide a detailed introduction of some representative UV-protection polymers, including UV-protective agent for cells, polymeric sunscreen, multifunctional UV-protective film, and UV-protective fabric. Among the works reviewed here, MCRs as chemical synthesis tools offer advantages such as high efficiency, a broad substrate scope, and modularity, and thus provide an effective strategy for synthesizing intrinsic UV-protective polymers and for simultaneously achieving biosafety, transparency, and functional diversity. The mild reaction condition of MCRs ensures their good compatibility with various polymer synthesis methods such as free radical polymerization, condensation polymerization, and post-polymerization modification, providing an effective approach for achieving large-scale manufacture of UV-protective polymers. Meanwhile, the molecular diversity of MCRs can effectively contribute to the development of materials with broad wavelength protection capabilities. Meanwhile, it should be acknowledged that the work reviewed above has seldom addressed preparation on an industrial scale, and that several of the systems described remain at the stage of proof of concept and are still some distance from practical production. The industrial-scale manufacture of UV-protective materials, reaction control, the reproducibility of radical polymerization and of surface modification, and the cost of the reactants deserve further investigations in this field. In the future, high-performance UV-resistant materials will remain a pursued goal in polymer research, and MCRs will continue to offer additional options for the efficient screening and utilization of UV-protective functional groups, for enhancing the UV-protective performance and transparency of film materials, and for improving the biosafety and environmental friendliness of sunscreens.

## Figures and Tables

**Table 1:** Summary of UV-protective polymers prepared by MCRs.

Product	Journal	Year	Ref.	MCRs	Synthesis Method	Mechanism	Performance *
UV-protective agent	J. Am. Chem. Soc.	2018	[40]	Biginelli	Direct polymerization	DHPM/DHPMT Functional-group introduction	47–74% cell viability ROS scavenging DNA protection
	Chem. Mater.	2022	[41]	Ugi	Direct polymerization	Functional-group introduction	>85% ROS scavenging 100% zebrafish hatching and 0% malformation
Polymeric sunscreen	Cell Rep. Phys. Sci.	2023	[42]	Biginelli	Direct polymerization	DHPM/DHPMT	High UVA absorption Environmentally friendly Superior in vivo protection to commercial sunscreen
	ACS Macro Lett.	2025	[43]	Biginelli	Direct polymerization	DHPMT Functional-group introduction	>70% cell viability
	Food Hydrocoll.	2022	[44]	Hantzsch	Post-polymerization modification	1,4-DHP Functional-group introduction	>88% DPPH scavenging Antibacterial
	Nat. Commun.	2026	[45]	Hantzsch	Post-polymerization modification	1,4-DHP Functional-group introduction	SPF ~80 84% visible transparency High UVA/UVB blocking >90% cell viability
UV-protective film	Mater. Horiz.	2022	[46]	Hantzsch	Surface functionalization	1,4-DHP	~100% UVA/UVB blocking ~90% visible transparency Hydrophobic property Long service life
	Cell Rep. Phys. Sci.	2024	[47]	Hantzsch	Post-polymerization modification	1,4-DHP Functional-group introduction	~99% UVA/UVB blocking 93% visible transparency Painting protected for 168 h
UV-protective fabric	ACS Appl. Mater. Interfaces	2022	[48]	Hantzsch	Surface functionalization	1,4-DHP	UPF ~69.2 <2.5% UV transmittance Hydrophobic property Washability
	Chem. Eng. J.	2023	[49]	Mannich	Surface functionalization	Functional-group introduction	High UV blocking 82.1% visible transparency 99% antimicrobial activity Washability
Dynamic material	Nat. Commun.	2025	[50]	Biginelli	Direct polymerization	DHPMT Functional-group introduction	>99% UV blocking 87% visible transparency Self-healing at 25 °C with UV blocking restored

* The performance is related to various factors such as the specific addition amount in the research, the thickness of the material, etc.

## Data Availability

No new data were created or analyzed in this study. Data sharing is not applicable to this article.
